# Nipah Virus Infects Specific Subsets of Porcine Peripheral Blood Mononuclear Cells

**DOI:** 10.1371/journal.pone.0030855

**Published:** 2012-01-27

**Authors:** Beata Stachowiak, Hana M. Weingartl

**Affiliations:** 1 Department of Medical Microbiology, Faculty of Medicine, University of Manitoba, Winnipeg, Canada; 2 National Centre for Animal Disease (NCFAD), Canadian Food Inspection Agency, Winnipeg, Canada; Blood Systems Research Institute, United States of America

## Abstract

Nipah virus (NiV), a zoonotic paramyxovirus, is highly contagious in swine, and can cause fatal infections in humans following transmission from the swine host. The main viral targets in both species are the respiratory and central nervous systems, with viremia implicated as a mode of dissemination of NiV throughout the host. The presented work focused on the role of peripheral blood mononuclear cells (PBMC) in the viremic spread of the virus in the swine host. B lymphocytes, CD4−CD8−, as well as CD4+CD8− T lymphocytes were not permissive to NiV, and expansion of the CD4+CD8− cells early post infection was consistent with functional humoral response to NiV infection observed in swine. In contrast, significant drop in the CD4+CD8− T cell frequency was observed in piglets which succumbed to the experimental infection, supporting the hypothesis that antibody development is the critical component of the protective immune response. Productive viral replication was detected in monocytes, CD6+CD8+ T lymphocytes and NK cells by recovery of infectious virus in the cell supernatants. Virus replication was supported by detection of the structural N and the non-structural C proteins or by detection of genomic RNA increase in the infected cells. Infection of T cells carrying CD6 marker, a strong ligand for the activated leukocyte cell adhesion molecule ALCAM (CD166) highly expressed on the microvascular endothelial cell of the blood-air and the blood-brain barrier may explain NiV preferential tropism for small blood vessels of the lung and brain.

## Introduction

Nipah virus is a zoonotic, highly pathogenic, biosafety level 4 (BSL4) virus within the family *Paramyxoviridae*, genus *Henipavirus* [1]. Humans infected with NiV suffer primarily from severe encephalitis with pulmonary involvement in high percentage of patients, and with fatal outcome in about 40 or more percent of laboratory confirmed cases, depending on the outbreak [2], [3]. All human cases during the initial 1998–1999 outbreak in Malaysia and Singapore were due to transmission of NiV from infected pigs [4], [5]. In Bangladesh, transmission of the virus from its natural reservoir the *Pteropus* bats to humans is by ingestion of contaminated date palm sap or fruit. Only one cluster of cases was thought to be due to transmission from pigs. In addition, nosocomial and human to human transmission were also reported [6].

Better understanding of the NiV infection in swine would be critical for developing control measures, should another NiV disease outbreak initiate in swine. Although infection ratio in affected swine herds approached in the Malaysian outbreak 100%, morbidity varied based on age, and mortality rate was rather low (1–5%). The disease in pigs was mainly respiratory with involvement of a central nervous system in some animals [5]. Suspected viremic dissemination of NiV throughout the swine host was confirmed during the experimental infections of pigs [7]. The low level viremia is both, cell free and cell associated. Involvement of the immune cells was suggested by virus RNA detection in peripheral blood mononuclear cells (PBMC) of NiV infected pigs, and by positive staining for NiV antigen in lymphocytes and mononuclear cells within lymph nodes and spleen, accompanied by lymphocyte necrosis and later in the infection also by lymphoid depletion in the lymph nodes [8], [9], [10]. *In vitro* infection of PBMC showed that NiV antigen was present in monocytes and a subpopulation of lymphocytes [10]. NiV antigen was also detected in infiltrating lymphocytes and monocytes of the perivascular cuffs in brain and respiratory system, although to a lower extent then in the endothelial cells of small blood vessels [9]. Infection and damage of the endothelial cells of small blood vessels, associated with vasculitis, is an important feature of NiV infection in susceptible species [11]. Interestingly, infection of large blood vessels was not detected.

Clinical outcome of Nipah virus infection in experimentally inoculated pigs (commercial Landrace cross breed) somewhat differs from the disease observed in the field in Malaysia. The infection is in majority of pigs asymptomatic or with mild respiratory signs compared to naturally infected pigs. However the nasal, oro-nasal or subcutaneous inoculations lead in more animals to severe central nervous signs compared to the field reports [7], [9], [10], [12]. In the nasally or oro-nasally infected piglets, NiV invades the central nervous system (CNS) directly from the oronasal cavity via cranial nerves, and by crossing the blood-brain barrier following viremic spread [9], [11]. In our attempts to produce positive control immune serum for diagnostic purposes, only 11 out of 16 piglets nasally infected with NiV survived until 4 weeks post infection, indicating 35% mortality rate in this experimental model. Four of the euthanized piglets, had not only NiV in the cerebrospinal fluid (CSF), but also bacteria considered to be associated with immune-compromised state (*Enterococcus faecalis*, *Streptococcus suis*) ([10] unpublished data). It appears that infection of pigs with NiV may predispose some of the pigs, possibly depending on the genetic variability in MHC (SLA I and SLA II), T cell receptors or cytokine genes [13] to secondary bacterial infections.

The aim of this study was to confirm the cell associated viremic spread of NiV in porcine host by determining permissibility of individual subpopulations of PBMC to the virus, and to determine possible additional effects on cells of immune system present in peripheral blood of the porcine host. The primary focus of the *in vivo* work was the early stage post infection (up to 7 dpi) to better understand mechanisms involved in pathogenesis of NiV in porcine host during the acute phase of the disease.

## Results

Viremia was implicated as a mode of dissemination of NiV throughout the host, including swine [7], [9], [14]. In order to determine the role of cells in the viremic spread of the virus, and permissiveness of porcine immune cells to Nipah virus, the work was initiated using PBMC. Due to constraints of work in BSL4 containment, selection and combinations of markers/antibodies had to be limited to maximum triple staining when internal staining for viral antigen was employed, and to only double staining for the cell surface markers.

### NiV infection of porcine PBMC subpopulations *in vitro*

**Figure 1:**
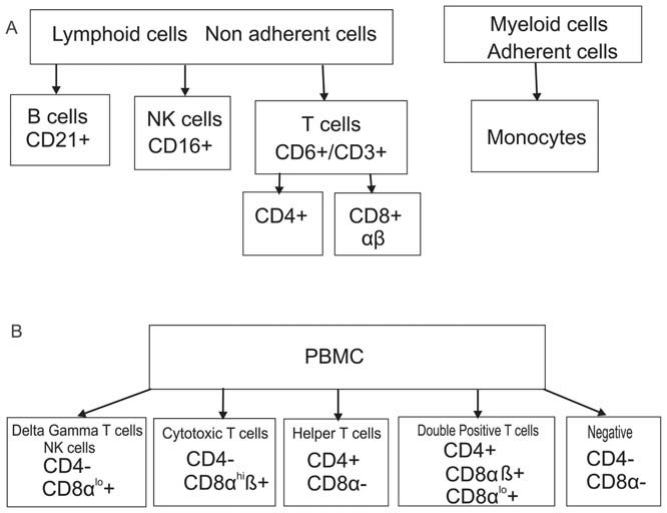
Flow chart of sorting and staining of PBMC. Sorting of cell subpopulations from peripheral blood mononuclear cells (PBMC) by magnetic beads coated with antibodies against selected markers (**Fig. 1A**), and staining of PBMC for CD4 and CD8 markers for analysis by flow cytometry (**Fig. 1B**). (^hi^) indicates high density expression of a specific marker, and (^lo^) indicates low density expression.

PBMC from non-infected pigs were separated into subpopulations prior to the inoculation. After adhering the monocytes to a cell culture plate, the non-adherent cells were sorted using magnetic beads coated with antibodies against CD16 (NK cells), CD6 (T lymphocytes), or CD21 (B lymphocytes) (**Figure 1.A**). The purity of NK, T, and B cells was verified by flow cytometry using the above markers. The T cells had the highest purity (90–95%), followed by NK cells (85%), whereas purity of B cells was the lowest in the range of 75–85%. The PBMC were alternatively sorted using CD3 marker and CD4 or CD8 markers, as this increased the purity of the T cell preparations in the double antibody sorting with the resulting purity around 95%. Initially CD6 marker was tested, but it appears that there was interference in binding between the anti-CD6 and the anti-CD4 or anti-CD8 antibodies. Sorted cells were inoculated with NiV in non-stimulated state, and for some of the cell preparations also after stimulation with PMA combined with ionomycin to approximate activated immune system post infection.

Replication of NiV in the individual subsets of cells was determined by detection of infectious virus particles in supernatant harvested from monocytes, NK and T lymphocytes. The multiplicity of infection (moi) in these experiments was based on NiV infectivity in Vero 76 cells to ascertain that the same concentration of infectious particles was used for inoculation of all cell preparations. The titre of the same NiV stock can be very different when titrated on Vero 76 cells and on immortalized alveolar macrophages (IPAM): 4×10^6^ and 5×10^4^ pfu/ml, respectively. Consequently, it was expected that actual moi can be as low as 0.001 (for example on monocytes).

**Figure 2:**
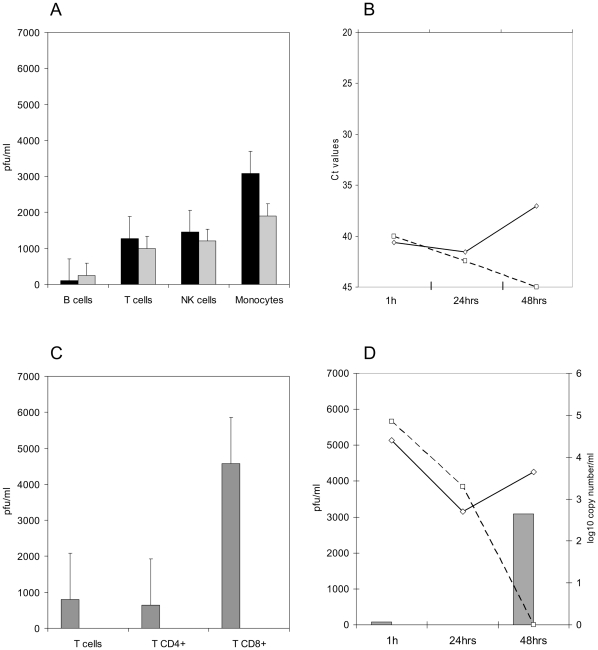
Replication of NiV in the individual subpopulations of PBMC. Sorted cells were inoculated with NiV in three independent experiments. Total of 10^5^cells per subpopulation were inoculated with 10^4^ PFU of NiV in one 1 ml volume. The infectivity in cell supernatants was determined by plaque titration on Vero 76 cells. **Fig. 2.A** Comparison of virus yield from stimulated and non-stimulated PBMC subpopulations at 48 hrs post inoculation. Black filled columns represent mean of pfu/ml in supernatant collected from non-stimulated cells, the grey filled columns represent mean of pfu/ml in supernatant collected from stimulated lymphocytes or monocytes. Purity of the individual cell preparations was in the following range: B cells - 75 to 85%, CD6+ T cells - 90 to 95%, CD4+ T cells - 85%, CD3+CD8+ T cells - 95%, NK cells – 80 to 85%, monocytes - close to 100%. **Fig. 2.B** Presence of genomic RNA at indicated time points post inoculation in cell lysates from monocytes inoculated with live NiV (solid line) or with gamma-irradiated NiV (dashed line). **Fig. 2.C** NiV yield in supernatants of sorted T lymphocytes at 24 hrs post inoculation. **Fig. 2.D** Detection of infectivity in supernatant harvested from monocytes at 24 and 48 hrs post inoculation with NiV (gray columns), and detection of viral RNA in cells inoculated with live virus (solid black line) or with gamma-irradiated NiV (black dashed line).

At 48 hrs, the highest virus yield was detected in supernatants harvested from monocytes (**Figure 2.A**). Very low level of infectivity recovered from the B cells was likely due to contaminating cells from other permissive subpopulations of PBMC (about 25–30%). Stimulation of cells did not significantly change the virus yield obtained in their supernatants, and was not used in follow up experiments.

At 24 hrs post inoculation, the highest infectivity was recovered from supernatants of the CD3+CD8+ sorted T lymphocytes (**Figure 2.B**). Lower yield of virus was recovered from the CD6+ T lymphocytes and the CD4+ T lymphocytes, corresponding with the observation that only lymphocytes carrying CD8+ marker were permissive to NiV infection (see flow cytometry results below). Interestingly, no infectivity was recovered from monocyte supernatants at 24 hrs post inoculation (**Figure 2.C**). The finding was supported by detection of NiV genomic RNA at different time points post inoculation. The amount of genomic RNA dropped at 24 hrs, and then significantly increased at 48 hrs post infection (**Figure 2.D**), while genomic RNA of gamma-irradiated NiV was gradually decreasing below detectable level by 48 hrs post exposure. Due to phagocytic nature of monocytes, most of the virions are likely digested or processed for antigen presentation, and only small number of infectious virions is able to initiate productive infection in these cells.

Expression of one of the NiV receptors, ephrin-B2 was determined to assess whether the block for virus replication could be at the level of virus attachment. In resting state, all T lymphocytes expressed ephrin-B2 mRNA, whether they were permissive to NiV or not (**Table 1**). On the other hand, monocytes and NK lymphocytes were permissive to NiV infection in resting state without expressing the mRNA of ephrin- B2, and started to express the mRNA upon stimulation and/or NiV infection. While stimulation of the B lymphocytes with PMA/ionomycin lead to ephrin-B2 expression, it did not render the cells permissive to NiV.

**Table 1:** Detection of mRNA expression of porcine ephrin-B2 in NiV inoculated and/or activated monocytes and enriched lymphocytes 48 hrs post inoculation.

Cell Subsets	Untreated cells	PMA/ionomycin treated cells	NiV inoculated cells	PMA/ionomycin treated, NiV inoculated cells
Monocytes	−	+	+	+
CD6+ T cells	+	+	+	+
CD3+CD8+ T cells	+	N/D	+	N/D
CD4+ T cells	+	N/D	+	N/D
CD21+ B cells	−	+	−	+
CD16+ NK cells	−	+	+	+

The (**+**) indicates mRNA of ephrin-B2 was detected. The (**−**) indicates mRNA of ephrin -B2 was undetectable. The (**N/D**) indicates that ephrin-B2 was not determined for this cell population.

### Infection of T lymphocytes with NiV

**Figure 3:**
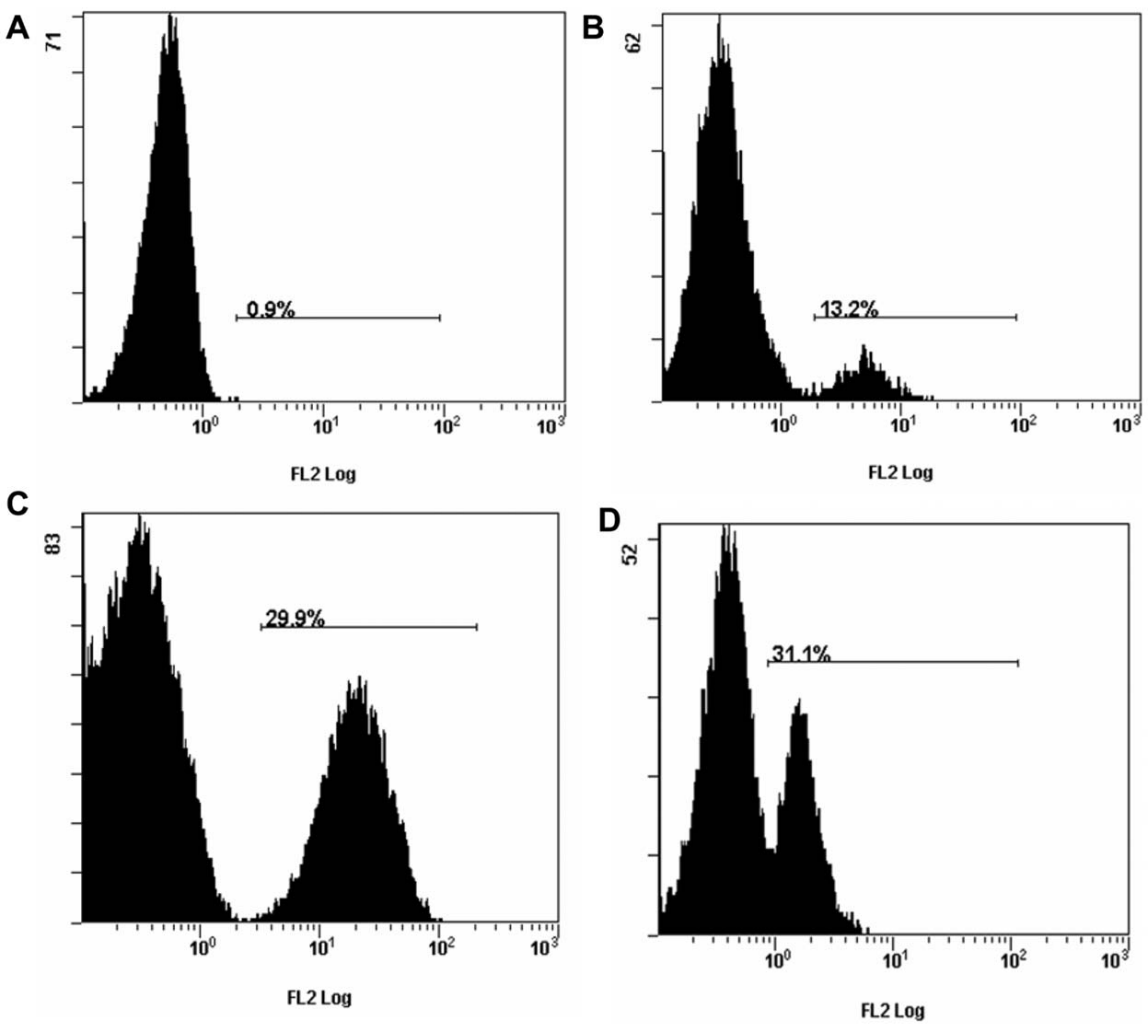
Intracellular staining for NiV nucleocapsid N or non-structural C protein in T cells. The cells were sorted based on the presence of CD4, CD6 or CD3CD8 markers, and infected with NiV at 0.1 moi. Representative flow cytometry histograms were selected from three independent experiments. **Fig. 3.A** Lack of intracellular staining for NiV non-structural C protein in CD4+ T cells at 24 hrs post inoculation. **Fig. 3.B** Intracellular staining for NiV C protein in CD3+CD8+ T cells at 24 hrs post inoculation. At 48 hrs post inoculation/infection about 30% of the CD3+CD8+ stained internally for the NiV C protein (**Fig. 3.D**), confirmed by about 30% of CD6+ T cells stained internally for the NiV nucleocapsid N protein (**Fig. 3.C**).

**Figure 4:**
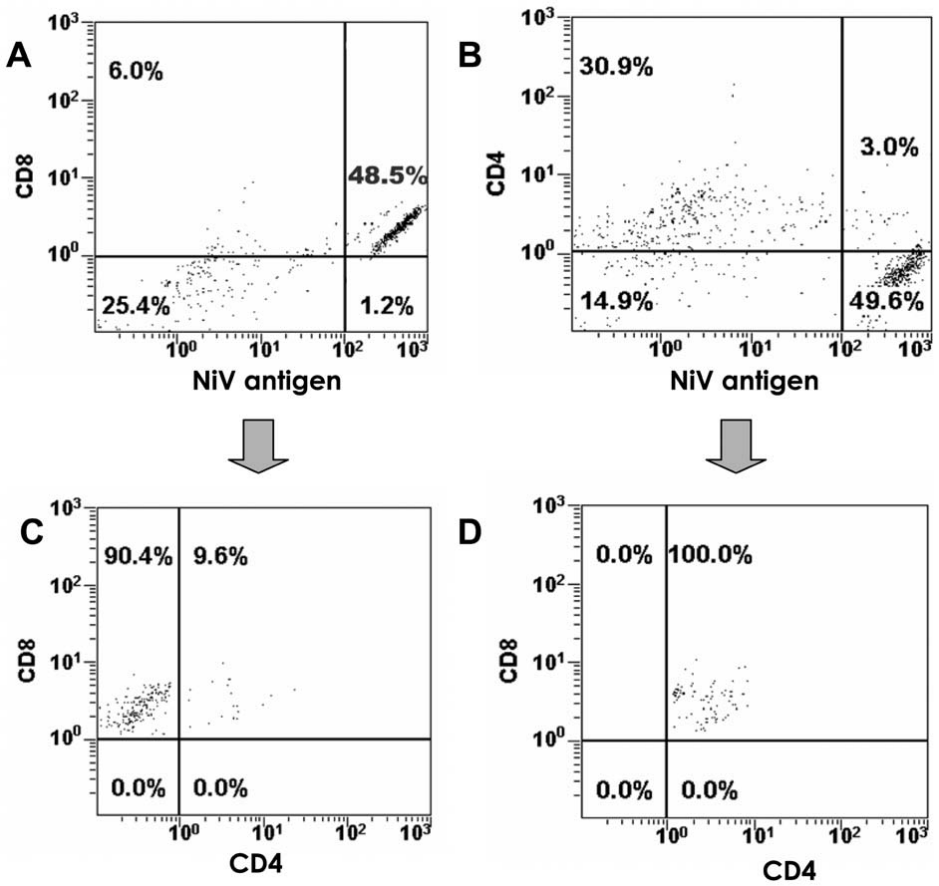
NiV infected CD6+ T cells triple stained for CD4 and CD8 markers, and NiV antigen. Dot plot of flow cytometry analysis of NiV infected CD6+ T cells, triple stained with monoclonal antibodies against CD4 and CD8 markers, and NiV polyclonal guinea pig antiserum 24 hrs post infection. T cells gated for CD8+ and NiV antigen (**Fig. 4.A**). T cells gated for CD4+ and NiV staining (**Fig. 4.B**). Analysis of cells positively stained for CD8+ and NiV antigen indicated that only low percentage (about 10%) was also positive for CD4 marker (**Fig. 4.C**). Analysis of cells positively stained for CD4+ and NiV antigen indicated that they were all positive for CD8 (**Fig. 4.D**) marker. In summary, cells with CD8 marker, whether CD4+CD8+ or CD4−CD8+ were the ones staining also for NiV antigen.

Infection of T cells with NiV was supported by internal positive staining for the NiV-N protein in the CD6+ sorted cells as analyzed by flow cytometry, and in the CD3+CD8+ sorted cells by positive staining for the non-structural C protein (**Figure 3**). NiV N antigen was detected in approximately 30% of CD6+ T cells at 48 hrs post inoculation at 0.1 moi. NiV C protein was detected in about 10% of the CD3+CD8+ T lymphocytes at 24 hrs post inoculation, and reached 30% at 48 hrs. The intensity of staining cannot be directly correlated, as NiV infected cells produce substantially lower amount of C protein compare to the N protein [15]. We were able to narrow the infection of the CD6+ lymphocytes to CD4−CD8+ and CD4+CD8+ T lymphocytes using triple staining for NiV antigen, CD8 marker, and CD4 marker (**Figure 4**): T cells gated for CD8+ and NiV antigen staining had high percentage of cells with positive staining for CD8+ and NiV (**Figure 4.A**). In contrast, T cells gated for CD4+ and NiV staining have only low percentage of double stained cells positive for both, CD4+, and NiV antigen (**Figure 4.B**). Analysis of cells positively stained for CD8+ and NiV antigen indicated that only low percentage (about 10%) was also positive for CD4 marker (**Figure 4.C**). Further analysis of cells positively stained for CD4+ and NiV antigen indicated that they were all positive for CD8 (**Figure 4.D**) marker. CD4+CD8− cells, and CD4−CD8− cells appeared to be non-permissive to NiV infection.

**Figure 5:**
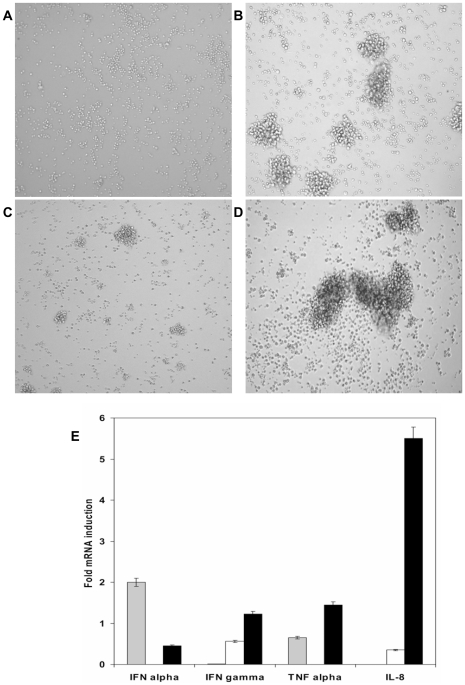
Stimulation of T cells with PMA/ionomycin and/or NiV infection. **Figs. 5.A–5.D** illustrate changes in cell appearance of the CD6+ T cells after 18 hrs post infection with NiV at 0.1 moi. Formation of cell clusters in NiV infected cells suggests that the infection stimulates T lymphocytes. Appearance of non-stimulated T cells (**Fig. 5.A**) in comparison to NiV infected T cells (**Fig. 5.B**) and the PMA/ionomycin stimulated cells (**Fig. 5.C**). NiV infected PMA/ionomycin activated T cells (**Fig. 5.D**). **Fig. 5.E** Cytokine RNA profiles were determined 48 hrs post incubation/infection by quantitative RT-PCR. Gray columns represent ratio of PMA/ionomycin activated cells and non-stimulated T cells; white columns represent ratio of NiV infected cells compare to non-stimulated cells; black columns represent ratio of infected PMA/ionomycin activated cells compared to PMA/ionomycin activated T cells.

Based on the cell culture images, NiV infection appeared to activate the T lymphocytes (**Figure 5.A–D**). Generally, formation of the cell clusters is considered to be a phenotypic characteristic of activation. To confirm the observation, changes in levels of selected cytokines were determined by semi-quantitative real-time RT-PCR in CD6+ cells. Relative levels of mRNA expression of IL-8, TNF α, IFN α and IFN γ are summarized in **Figure 5.E**. NiV infected cells slightly (less then one-fold) upregulated expression of IFN γ and IL-8 in non-stimulated cells. Significant upregulation of IL-8 expression was however observed in stimulated cells infected with NiV (6 fold). Upregulation of IFN γ and TNF α greater then one-fold was observed in stimulated, NiV infected cells. On the other hand, infection of lymphocytes with NiV appeared to down-regulate IFN α mRNA, both in non-stimulated and stimulated cells, when compared to the constitutive expression levels of IFN α in non-stimulated T lymphocytes or PMA/ionomycin activated T cells, respectively.

### Effect of NiV infection *in vitro* on PBMC

**Figure 6:**
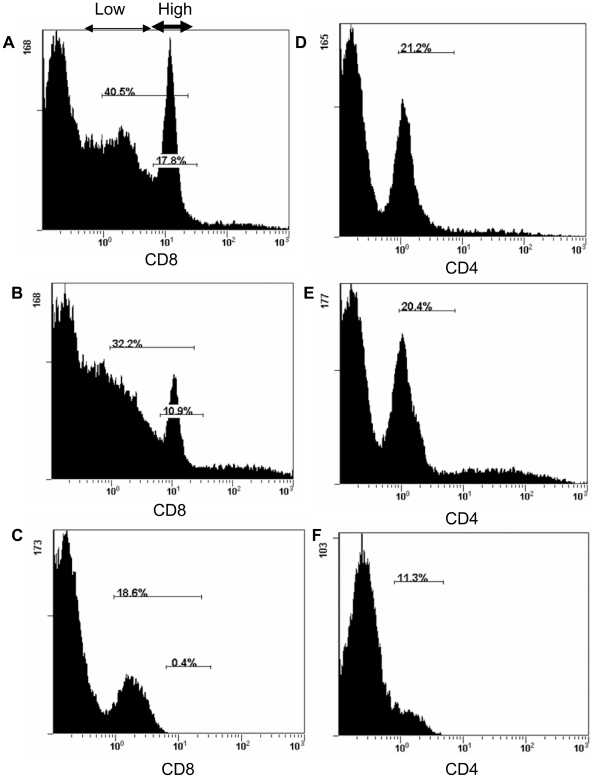
Changes in population frequencies of CD8+ and CD4+ cells following NiV *in vitro* inoculation of PBMC. **Fig. 6.A** PBMC control after 24 hrs incubation stained for CD8 marker. Thin arrow on top of the figure indicates CD8 αβ^lo^ T cells. The highest intensity peak corresponds to CD8 αβ^hi^ T cells (cytolytic T cells) indicated by the bold arrow. **Fig. 6.B** NiV infected PBMC stained for CD8 marker at 24 hrs post inoculation. **Fig. 6.C** At 48 hrs post inoculation, the peak corresponding with CD8 αβ^hi^ T cells was absent, and the proportion of CD8+ cells decreased from 40% to about 20%. **Fig. 6.D** PBMC control stained for the CD4 marker after 24 hrs incubation. **Fig. 6.E** Cells inoculated with NiV stained at 24 hrs post inoculation for CD4 marker. **Fig. 6.F** Decrease in CD4+ cells from 20% in the control uninfected PBMC to about 10% at 48 hrs post inoculation with NiV.

Effect of NiV infection on lymphocyte population frequencies was studied in the context of PBMC to approximate the *in vivo* situation, allowing for interaction (cell cross-talk) of several cell populations. We were able to follow up the lymphocytes staining for CD4 or CD8 marker (**Figure 1.B**). At 24 hours post infection the number of CD8+ cells decreased from about 40% to 30% and down to 20% at 48 hrs post infection, with notable depletion of the CD8+^hi^ cytolytic T cells (**Figure 6.A–C**). Almost no change in percentage of the CD4+ population was observed at 24 hrs post inoculation (**Figure 6. E**), while apparent decrease in CD4+ was observed at 48 hrs post infection (**Figure 6. F**). The drop in CD4+ cells could be attributed to the decrease in CD4+CD8+ cells (mostly memory helper T cells), however it cannot be excluded that the significant drop at 48 hrs was due to bystander cell death of the CD4+CD8− cells, considering the *in vivo* data below.

### Effect of NiV infection *in vivo* on PBMC

**Figure 7:**
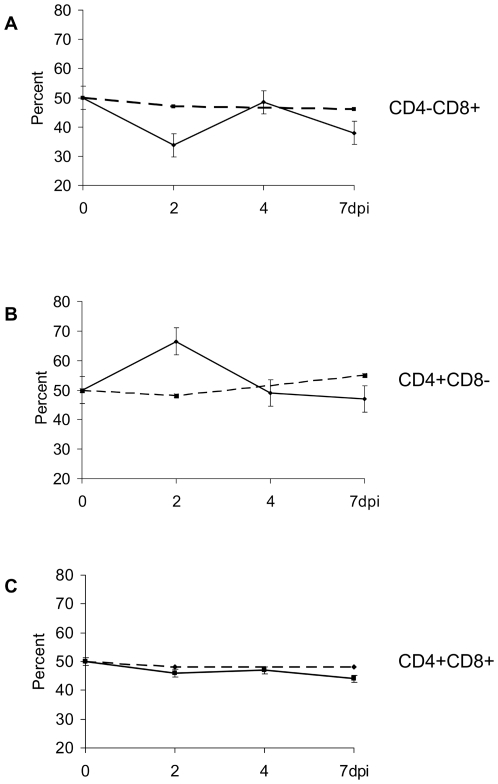
T cell subpopulation frequencies in NiV infected pigs during the acute phase of the infection. Changes in CD4D8 cell subpopulation frequencies in PBMC of pigs infected with NiV based on flow cytometry analysis. The values obtained at 0 dpi were arbitrarily set at 50%. The data (mean and standard error) are based on six infected (solid line) and 4 control (dashed line) animals. Notably, significant changes with opposite trends were observed for CD4−CD8+ T cells (**Fig. 7.A**), and CD4+ CD8− T cells (**Fig. 7.B**). The changes in CD4+CD8+ T cell frequency, although statistically significant, were only minor, perhaps with slight decline toward the 7 dpi in the infected piglets compared to the controls (**Fig. 7.C**).

Low levels of NiV RNA were detected in PBMC from 2 to 7 days post inoculation by real-time RT-PCR targeting the N gene, corresponding with previously published data on low level of NiV detection in serum, PBMC or whole blood in swine [7], [9], [10]. Although it was difficult to detect the virus in peripheral blood of NiV infected piglets by flow cytometry, and total white blood cell count was considered to be within the normal range (data not shown), an effect on population frequencies of lymphocytes was observed using double staining for CD4 and CD8 surface markers. At two days post inoculation a significant drop (p>0.05) in CD4−CD8+ lymphocytes was observed in PBMC collected from all 6 infected pigs compared to 4 control animals (**Figure 7.A**). At the same time point, the CD4+CD8− population circulating in peripheral blood increased significantly (p>0.05) when evaluating all infected piglets versus negative control pigs (**Figure 7.B**). At 4 dpi, both subpopulations returned close to basal level. The population frequency of double positive (CD4+CD8+) T cells did not change substantially compared to non-infected pigs. Low gradual decrease following the inoculation was statistically significant (p<0.05) when compared to control animals and the pre-infection status, but may not have a biological significance (**Figure 7.C**).

**Figure 8:**
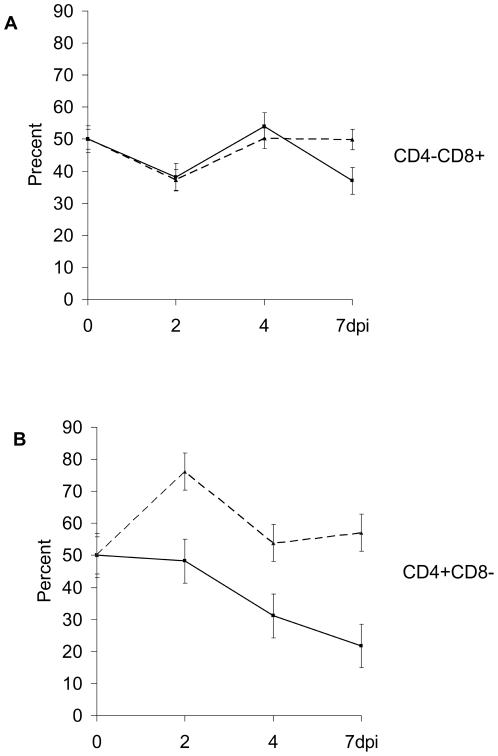
Comparison of T cell subpopulations between pigs that died during acute infection versus survivors. The differences in CD4+ and CD8+ cell subpopulations during the acute infection with NiV up to 7 dpi. Values, based on flow cytometry analysis, were arbitrarily set as 50% at 0 dpi. The solid line represents piglets that died at 7 dpi, the dashed line represents the survivors. Standard Error is represented as error bars for 2 pigs per each group/mean value. Survivors had significantly higher values for the CD4−CD8+ (**Fig. 8.A**) at 7 dpi compare to the piglets which died at that day. Marked difference was observed for the CD4+CD8− T lymphocytes (**Fig. 8.B**). The down-trend starting almost immediately post infection for this cell subpopulation in pigs that died due to NiV infection was especially pronounced. In contrast, there was an up-regulation of CD4+CD8− T helper cells at 2 dpi in the survivors.

Interesting trends appeared in population frequencies for piglets which had to be euthanized during the first week post inoculation compared to piglets which survived past 7 dpi, and were euthanized at 28 dpi (end of the study). In piglets requiring early euthanasia, the CD4−CD8+ T cells dropped again at day 6/7, in contrast to the survivors, where the CD4−CD8+ T cells returned to pre-infection levels (**Figure 8.A**). Dramatic difference in population frequency of the CD4+CD8− lymphocytes was observed between survivors and non-survivors. While there was a significant increase of CD4+CD8− T helper cells in survivors at 2 dpi, returning to normal by 7 dpi, piglets which had to be euthanized had no upregulation of CD4+CD8− T cells, and the population frequency continued to decrease until the end point at 7 dpi (**Figure 8 B**).

## Discussion

We have previously determined that monocytes and a subset of lymphocytes had internal staining for NiV antigen within the infected PBMC [10].The experimental work presented in this manuscript confirmed that NiV can productively infect porcine peripheral blood monocytes. Increased levels of NiV genomic RNA were detected in infected monocytes at 48 hrs post infection, while no viral RNA was detected in cells inoculated with gamma-irradiated NiV. Interestingly the amount of genomic RNA decreased in the infected monocytes at 24 hrs post inoculation, before increasing to the levels higher then one hour post inoculation. Correspondingly, no infectivity was detected in the supernatants harvested from the cells at 24 hrs post inoculation, and relatively high virus yield was obtained at 48 hrs post infection. The dynamics of NiV replicaiton in monocytes would indicate that majority of the virus is phagocytosed, and only small proportion of the virus is internalized in an alternative way allowing replication.

Here we determined that the NK cells (also carrying the CD8+ marker) and the CD8+ subset of T lymphocytes were permissive to NiV, thus confirming the hypothesis of cell associated viremia during NiV infection in the swine host. The level of NiV viremia detected in the peripheral blood was low but comparable to previously published work in pigs and other species [7], [9], [16]. This phenomenon is not exceptional for paramyxoviruses, and was observed e.g. for measles virus in humans [17]. The low viremia somewhat reflects the type of cells infected in the swine host, where monocytes and NK cells altogether represent between 5–15% of PBMC, and the majority of CD8+ cells home into the lymph nodes via lymphatic system [18]. The permissiveness of lymphocytes and monocytes to NiV infection appears to be species specific. Recently Mathieu et al. [19] reported that although leukocytes likely contribute to viremia in humans and hamsters, NiV does not replicate in them, and the cells serve merely as mechanic transporters.

Infection of different subpopulations of PBMC did not appear to be entirely dependent on pre-existing expression of ephrin-B2, a primary receptor for NiV [20], [21]. Resting monocytes and NK cells did not have detectable levels of ephrin-B2 mRNA, but upregulated the ephrin-B2 mRNA following stimulation and/or NiV inoculation. Based on literature, monocytes express the alternative ephrin-B3 receptor for NiV [22], sufficient for the initial infection. Mechanism of the initial infection of NK cells with NiV is not clear at this moment, as there are no reports on ephrin-B3 expression (or lack of thereof) in NK cells. However, since the NK cells preparation contained up to 25% of other cells permissive to and activated by NiV, the rapidly released proinflammatory cytokines [23] likely stimulated ephrin-B2 expression on the NK cells, and rendered them permissive to NiV. While all CD6+ T lymphocytes expressed ephrin-B2, only the ones expressing CD8 marker were permissive to NiV. Further investigation of this phenomenon could lead to elucidation of the role of CD8 marker in the permissiveness of porcine immune cells to NiV or identification of important cellular factors involved in block of NiV replication block in the CD6+ cells lacking the CD8 marker.

Infection of the immune cells can modulate the immune response by down regulation of specific cell populations due to virus replication followed by cell death or due to release of cytokines with subsequent bystander death of susceptible cells. In the context of *in vitro* infected PBMC, the CD8+ ^hi^ T cells were the most affected subset of cells already at 24 hrs post inoculation with almost complete elimination of this population at 48 hrs post infection, consistent with these cells being permissive to NiV. CD4−CD8+^hi^ T cells were characterized in pigs as cytotoxic T cells and their decrease would have an effect on clearance of virus infected cells [18]. We have observed decrease in CD4−CD8+ cell population frequency also *in vivo* at 2 dpi, however it remains to be determined whether those were NK cells, γδ T cells or cytotoxic T cells to offer any comments on the *in vivo* significance.

A decrease in CD4+CD8− T helper cells, observed *in vitro*, would influence development of humoral immunity. Interestingly, the work with *in vivo* NiV infected PBMC confirmed the dramatic decrease in population frequency of CD4+CD8− T cells in piglets which succumbed to NiV infection, while values for surviving piglets indicated that the CD4+CD8− T cell subset expanded following the infection, corresponding with functional humoral response. Although the data are limited, they highlight previous findings indicating that the development of humoral immunity is critical factor in protective immune response to NiV [24]. In surviving animals, the production of antibodies is not considered impaired, but may be somewhat delayed compared to development of antibodies against swine influenza or Hendra virus [10], [25]. It remains to be determined whether drop in CD4+CD8− T cell population can be used to predict the outcome of NiV infection in pigs.

Additional work is warranted to understand the role of NK cells, monocytes and plasmacytoid dendritic cells in the pathogenesis and development or modulation of immune response to NiV, which decides the outcome of the NiV infection or the secondary bacterial infections. In summary, it appears that the focus of further studies has to shift to innate immune response, as the critical aspect in the development of adaptive immune response. This is also supported by our finding of down-regulation of IFN α production during NiV infection in the activated CD6+ T cells. While *in vitro* production of IFN-type I was demonstrated in NiV infected endothelial cells, the infection down-regulated production of IFN-type I in several other cell lines of human origin, including neuronal M17 cells [26], [27]. Block of IFN α production by NiV infected lymphocytes, and possibly other immune cells – such as plasmacytoid dendritic cells or dendritic cells at the site of infection may have significant impact on immune response activation and regulation, and contribute to disease progression/pathogenesis in NiV infected host [28].

Our main interest was to better understand the role of peripheral immune blood cells in the viremic spread of NiV across the blood brain barrier (BBB) in the porcine host. Identification of the CD3+**CD6+**CD8+ T lymphocytes, as cells productively infected by NiV, and thus actively contributing to viremia was of a special interest. CD6 marker is a strong ligand for the activated leukocyte cell adhesion molecule (ALCAM - CD166) expressed on the microvascular endothelium of the blood brain barrier (BBB), and which is upregulated during the inflammation [29]. The ALCAM is also expressed on the microvascular endothelial cells of the blood-air barrier in lung [30]. Consequently, dissemination of NiV within the host by the CD3+**CD6+**CD8+ cells would be preferentially targeted to small blood vessels in these two organs. Infection and vasculitis were described in small blood vessels in several hosts, but not in the larger vessels [11], consistent with this hypothesis.

**Figure 9:**
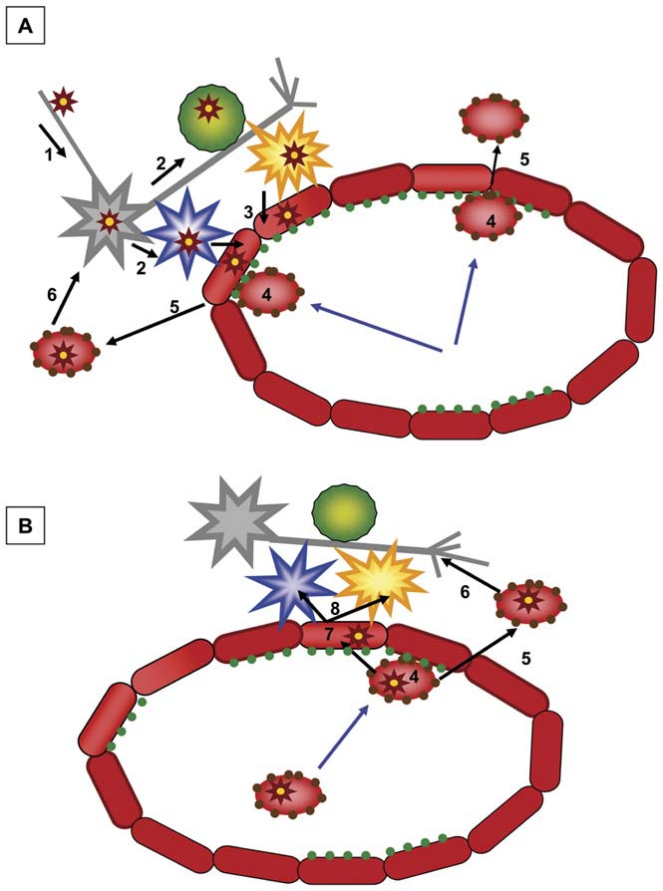
Proposed microvascular component of brain invasion by NiV in swine. **Fig.9.A**. NiV invasion of the central nervous system (CNS) initiated in the nasal cavity leads to virus spread into the brain via neurons [1]. NiV infects cells in contact with neurons: oligodendrocytes, astrocytes and microglia [2]. Astrocytes and microglia are in a direct contact with microvascular endothelial cells (MEC), allowing for direct NiV iter-cellular transmission and infection of the MEC [3]. Infected astrocytes, microglia and MEC recruit leukocytes by release of cytokines (blue arrows). From the incoming leukocytes, the CD6+ T cells can firmly attach to the MEC via the CD6-CD166/ALCAM interaction [4], form transmigration cup, and translocate into the brain parenchyma. (The NK cells and monocytes have low expression of the ALCAM on their surface, and can also transmigrate into the brain. Since the ALCAM-ALCAM interaction is weaker then CD6-ALCAM one, this may be a later event.) The translocating leukocytes can get infected during the transmigration due to intimate contact with NiV infected MEC [5], and spread the virus to the parenchymal cells in the brain [6], thus initiating the CNS invasion via BBB. **Fig. 9.B**. Alternatively, the recruited lymphocytes can be already infected with NiV. By forming the transmigration cap [4], they can during transmigration infect the MEC [7], spreading subsequently the virus to the in-contact astrocytes and microglia [8], and/or after transmigration across the BBB infect additional parenchymal cells [6]. This mechanism initiates the CNS infection, due to the virus crossing the BBB, and can also apply to invasion of the brain by NiV in other hosts then pigs. Gray star - neuron, blue star - astrocyte, green circle - oligodendrocytes, yellow star - microglia, small brown stars with yellow center - NiV, dark red ovals adjacent to each other - MEC forming the BBB, green dots - schematic representation of the ALCAM, light centered reddish ovals - T lymphocytes with CD6+ markers (dark brown dots), blue arrows - transmigration of leukocytes across the BBB, black arrows - NiV spread.

Based on previous work of our group, reports in the literature, and findings reported in this manuscript, we are proposing more detailed mechanism for the NiV neuroinvasion in swine host following nasal inoculation. **Figure 9** summarizes the events in the vicinity of a small blood vessel.

The virus gains access to the brain directly from the nasal cavity by infecting naked nerve endings (cilia) of the olfactory receptor cells (neurons) located in the mucosa of the olfactory epithelium, and the infection moves in a retrograde direction into the brain. In addition, infection of the nasal respiratory epithelium and of the basal cells of the olfactory epithelium leads to infection of the trigeminal nerve endings, and proceeds through the trigeminal ganglion into the brain. Viral antigen was detected in neurons, oligodendrocytes, astrocytes and microglia [9] with the later three cell types presumably infected by virus transmission from neurons at the very early stage. Astrocytes (possibly participating in formation of BBB) and microglia being in direct physical contact with capillaries transmit the virus to the endothelial cells. NiV infected endothelial cells produce IL-8 beside other inflammatory cytokines [27] which would recruit T-lymphocytes and monocytes. In our current work, the T lymphocytes also produced IL-8 upon infection, and would be likely contributing to the chemotaxis. In addition, neurons, astrocytes and microglia coordinate activation and regulation of local inflammatory reactions during infection, including release of cytokines and recruitment of monocytes and lymphocytes from circulation [31]. From the recruited cells, the CD6+ cells would be the ones most strongly adhering to the microvascular endothelial cells, forming transmigration complexes and moving into the brain parenchyma [29]. This interaction could lead to infection of CD6+CD8+ lymphocytes by the microvascular endothelium at the point of crossing the BBB. Alternatively, the endothelial cells can become infected by lymphocytes already replicating the virus, and attracted to the capillaries due to signalling by astrocytes or microglia. Lymphocytes were identified based on morphology as one of the antigen positive infiltrating cells associated with vasculitis in the brain [9], [14]. Beside the CD6+CD8+ lymphocytes, monocytes and NK cells can also transmigrate into the brain via weaker ALCAM - ALCAM interaction [29], [32]. At this point, NiV would start the viremia associated invasion of brain by crossing of the BBB from blood. Once the microvascular endothelial cells become infected, the cell free NiV in plasma may be able to cross the BBB as well [33].

The finding that a subtype of the T cells carrying CD6 surface marker contribute to viremia in NiV infected host would provide an explanation as to why the virus has a strong tropism for the microvascular endothelial cell of the blood-air and the blood-brain barrier, providing better understanding of the pathogenesis of NiV in general.

## Materials and Methods

### Viruses

Nipah virus re-isolated at NCFAD from pigs infected with human isolate (kindly provided by T. Ksiazek and P. Rollin, CDC, Atlanta) was used for animal inoculation. The *in vitro* work was done initially in parallel with both, the NCFAD swine isolate and the CDC human isolate (passage 5 in Vero 76 cells).

### Virus plaque titration

Vero 76 cells were grown to 100% confluency in 6 well plates (Costar, Corning Inc. Corning, NY). The cells were washed, and 400 µl of samples serially diluted from 10^−1^ to 10^−4^ in DMEM were added to duplicate wells. Inoculum was removed after one hour incubation, and replaced with 1.5 ml of 2% carboxymethycellulose (Sigma-Aldrich, Oakville, ON) in DMEM/2% FBS overlay. After 48 hrs incubation period, cells were fixed by the addition of 10% PBS buffered formalin (Fisher Scientific, Ottawa, ON, CA), and stained with crystal violet (0.1% w/v crystal violet in H_2_O). The titre of the virus was determined as plaque forming units per ml (PFU/ml).

### Cells

Vero 76 cells (African green monkey kidney, epithelial) (ATCC) were cultured in Dulbecco's Modified Eagles Medium (DMEM), supplemented with 10% fetal bovine serum (FBS) (Wisent Inc., St. Bruno, Que, CA). IPAM (immortalized porcine alveolar macrophage) cells 3D4/31 grown in RPMI 1640 media supplemented with L-glutamine and 10% FBS were used as positive control for intracellular detection of NiV [34].

Peripheral blood mononuclear cells (PBMC) for the *in vitro* studies were harvested from four pigs in the age group 10–15 weeks and six 40 weeks old pigs (Landrace cross) using cell preparation tubes, and following the protocol recommended by the manufacturer (Becton Dickinson, Oakville, ON, CA). The PBMC were resuspended in RPMI supplemented with 10% FBS, 10 mM Hepes, and 100 IU penicillin/100 µg/ml streptomycin (Wisent).

In the *in vivo* studies, PBMC were harvested as described above from nine piglets 4–6 weeks of age infected intranasally with 2.5×10^5^ PFU of NiV per animal. The PBMC were harvested under BSL4 conditions for the cell analysis prior to the inoculation, and on days 2–7 post inoculation. The blood from four control pigs was collected at the corresponding time points.

Ethics statement: The collection of blood from Nipah virus infected piglets was included as a component of the AUD ID#: C-10-002 (Production of positive and negative pig samples for the evaluation of diagnostic tests for Nipah virus infection). The collection of non-infected blood samples was included as a component of the AUD ID#: C-10-002, AUD ID#: C-09-002 (Ebola virus infection in pigs), AUD ID#: C-11-004 (Age sensitivity of pigs to Ebola virus and interspecies transmission), AUD ID#: C-09-011 (Novel H1N1 2009 Influenza transmission studies in swine). The above studies were approved by the Animal Care Committee of the Canadian Science Centre for Human and Animal Health. The collection of blood samples from the older pigs followed policy No. 1404, permit No. S09-008://, approved by the Animal Care Committee of the University of Manitoba. All animal manipulations strictly followed the guidelines of the Canadian Council on Animal Care.

### *In vitro* infection studies in subpopulations of PBMC

#### Cell separation into individual subpopulations of PBMC

Monocytes in the PBMC preparation were allowed to adhere onto 12 well plates (Costar) by overnight incubation at 37°C in a 5% CO_2_ incubator. The non-adherent cells were removed by light washing with PBS, pelleted by centrifugation at 300 g for 10 minutes prior to sorting by positive selection based on CD6+, CD16+, and CD21+ expression (**Figure 1A**). Cells were labelled with mouse monoclonal anti-human CD21 (diluted 1∶100) (IgG1, AbD SeroTec, Raleigh, NC) antibody to obtain B lymphocytes, or by mouse monoclonal anti- porcine CD16 (1∶100) (IgG1,AbD SeroTec) antibody to obtain NK cells, or with CD6 (1∶100) (IgG2a, BD Pharmingen, San Diego, CA) antibody to obtain T lymphocytes, and separated using paramagnetic microbeads coupled with rat anti-mouse IgG2a/b or goat anti-mouse IgG1 antibody (Miltenyi Biotech, Auburn, CA), respectively. Separation was performed in MACS buffer (phosphate buffered saline, 1% bovine serum albumin (BSA), 0.09% sodium azide) according to Miltenyi Biotech protocols. Briefly, cells were filtered through the pre-separation filter and loaded onto a ferric LS column in a magnetic field such that only labelled cells were retained in the column. Enriched cells were eluted with MACS buffer at a high flow rate by removing the magnet, washed twice with 5% FCS/ RPMI media by centrifugation at 300 g for 10 min, and plated onto12 well plates in the same medium.

Non-adherent cells were also used for separation of cells based on the expression of CD4 and CD8 markers. Cells were either selected for the CD4 marker using mouse monoclonal antibodies (mAb) diluted 1∶100 (IgG2bk, BD Pharmingen) or pre-selected for the CD3 marker using mAb against this marker diluted 1∶100 (IgG2ak, BD Pharmingen), followed by the CD8+ selection by mAb diluted 1∶100 (IgG2ak, BD Pharmingen). All separations were done using paramagnetic microbeads coupled with goat anti-mouse IgG1 (Miltenyi Biotech, Auburn, CA) as described above.

Before plating the cells, viability was determined by trypan blue exclusion method. Purity of recovered cells was determined by cytometric analysis of labelled cells using secondary goat anti-mouse Alexa 488 IgG_1_ (H&L) antibodies (Molecular Probes, Invitrogen, Burlington, ON, CA).

### NiV inoculation of sorted cells

The separated monocytes, CD21+, CD16+, CD6+, CD3+CD8+ and CD4+ cells were infected with NiV at 0.1 moi for 1 hr at 37°C, 5% CO_2_. The input cell number for separated cells was 10^5^ cells/well. The non-adherent cells were washed twice by centrifugation at 300 g for 10 min following the virus adsorption, and resuspended in the RPMI supplemented with 2.5% FBS, plated onto 12-well plates in 500 ml volumes, and incubated for 24 or 48 hrs at 37°C, 5% CO_2_. The monocytes were also washed twice prior to adding the same medium as for the lymphocytes. Stimulated cells were pre-treated for 1 hr prior to addition of NiV by ionomycin 430 ng/ml (Sigma - Aldrich, Oakville, ON, CA) and phorbol 12-myristate 13-acetate (PMA) 7 ng/ml (Sigma B Aldrich) in RPMI media. The incubation medium for the activated enriched cells was also supplemented with PMA/ionomycin. Supernatant and cells were harvested at 24 hrs and/or 48 hrs, depending on cell population, and plaque titrated on Vero 76 cells, using protocol described in 2.2.

In addition, adhered monocytes were trypsinized trypsinized with 0.25% trypsin/EDTA (Wisent) for 15 min at 37^N^C, 5% CO_2_, let to recover for 2 hrs at 4°C, and then inoculated in suspension either with NiV at 0.1 moi or with NiV gamma-irradiated by 5 MRADs at a dilution corresponding to 0.1 moi for 1 h at 37^N^C, 5% CO_2_. Following the incubation, cells were washed three times with PBS by centrifugation at 600 g for 5 min. Following the third wash, cells were resuspended in the RPMI/10% FCS, plated onto 12-well plates in 1 ml volumes of 10 ^5^ cells/well and incubated for 1 h, 24 and 48 hrs at 37^N^C, 5% CO_2_. Supernants and cells were collected at each time point. The supernatants were assayed for infectivity by plaque assay. The cells were trypsinized as described above, washed and collected by centrifugation. The pellets were used for detection of NiV genomic RNA, as described below.

### Intracellular staining for NiV antigen

CD6+ enriched T cells infected with NiV were stained intracellularly for NiV N antigen 48 hrs post infection using following protocol: The cells were fixed with 100 µl of BD Perm/Wash Cytofix/Cytoperm solution (BD Pharmigen) for 30 min at 4°C, washed twice, followed by addition of 1∶50 diluted mouse monoclonal anti-N antibody. After incubation on ice for 30 min, the cells were washed twice with BD Perm /Wash and secondary antibody was added. Anti-mouse Alexa 488 IgG diluted 1∶1000 and incubated with the cells in dark at 4°C for 30 min, washed twice with BD Perm/Wash. The cells were fixed in 4% formaldehyde for 24 hrs in order to remove them from the BSL4, and resuspended in 500 µl of MACS buffer for flow cytometry analysis.

The NiV infected CD6+ lymphocytes were also triple stained for the CD4, CD8 markers as described above, followed by permeabilization of the cells and staining for the NiV antigen using 1∶100 diluted guinea-pig polyclonal NiV antiserum and anti-guinea pig Alexa IgG 594 as secondary antibody, diluted 1∶1000.

The separated CD4+ cells, and the CD3+CD8+ cells infected with NiV were intracellularly stained with 1∶100 diluted rabbit polyclonal anti-C antibody, kindly provided by Dr. Chieko Kai (University of Tokyo), followed by secondary anti-rabbit Alexa IgG 568 antibody (diluted 1∶1000). All samples were analyzed on a two laser Flow Cytometer (Beckman Coulter). Cell populations of interest were gated for lymphocytes based on light scatter characteristics and counted to 10,000 events.

### Ephrin-B2 RT-PCR

Total cellular RNA was isolated by TriPure isolation reagent (Roche Diagnostics Corporation, Indianapolis, IN) as per manufacturer's protocol. RNA was heat inactivated for 10 min at 65°C prior to DNase treatment. RNA was treated with Turbo DNA Free DNase (Ambion, Austin, TX) according to the manufacturer's protocol. DNase treated RNA concentration was determined using 260/280 nm light absorbance ratio (N-D spectrometer Nano Drop Technologies). Reverse transcription was performed using Superscript II kit (Invitrogen). A total of 6 µl mRNA was added to each RT reaction and incubated for 45 min at 50°C followed by 72°C for 15 min. Two microliters of cDNA were added to Hotstart PCR mix (Qiagen, Mississauga, ON, CA) which included 10 µM of forward and reverse ephrin-B2 primers. A total 25 µl reaction was incubated for 15 min at 95°C followed by 35 cycles of (95°C -30 sec, 55°C -45 sec, 72°C -30 sec) and extension period of 10 min for 72°C. PCR fragments were run on 1.5% agarose gels with a 1 kp ladder (Invitrogen). Forward (ACCAGGCATAATGAGCCAAC) and reverse (CCTCAGCGGATGATAATGT ephrin-B2 primers were designed based on published porcine sequence (Accession NM0001114286 and EF682141.1) by using Primer 3 Input 4.0 software.

### Real-time semi-quantitative RT-PCR for mRNA cytokine expression

Total cellular RNA was extracted from enriched lymphocytes by TriPure isolation reagent (Roche Diagnostics) method. Reverse transcription was performed using random hexamer primers, and employing Quanitect kit (Qiagen). Ten microliters of 100 ng/µl samples were added to each RT reaction. After DNase treatment at 42°C for 4 min, RNA was reverse transcribed at 42°C for 20 min, and the reaction was terminated by 2 min incubation at 72°C. The real time PCR was performed using Taqman 3100 and Quantitech SYBR green PCR kit (Qiagen). Two microlitres of cDNA were added to a total volume of 25 µl. Real time PCR reaction had the following conditions for all primer sets (300 nM): 95°C for 15 min, followed by 41 cycles of (95°C - 15 sec, 55°C - 30 sec, 72°C - 30 sec). Primers used for amplification were following:

IL-8 (TTCTGCAGCTCTCTGTGAGGC/ GGTGGAAAGGTGTGGAATGC) [35],

TNF α (CCCCCAGAAGGAAGAGTTTC/CGGGCTTATCTGAGGTTTGA) [35],

IFN α (TCAGCTGCAATGCCATCTG/AGGGAGAGATTCTCCTCATTTGTG) [36],

IFN γ (TGGTAGCTCTGGGAAACTGAATG/GGCTTTGCGCTGGATCTG) [36], and cyclophilin (TAACCCCACCGTCTTCTT/TGCCATCCAACCACTCAG).

Cyclophilin primers were designed by Primer 3 Input 4.0 and porcine cyclophilin sequences obtained from BLAST search database. Dissociation curve and controls were added to each reaction. The real time RT-PCR reactions were performed in duplicates. A relative expression ratio of cytokine gene was calculated 2^−∧∧Ct^ according to Livak and Schmittgen [37], and using cyclophilin mRNA as reference.

### Real-time RT-PCR for NiV RNA detection

Real-time RT-PCR targeting NiV N gene was performed as described previously by Guillaime et al. [38]. All cultures (PBMC, enriched T cells, enriched B cells, enriched NK cells, IPAM and VERO 76 cells) used in the *in vitro* study for NiV inoculation were verified for NiV infection by NiV N-gene RT-PCR. In the *in vivo* study, detection of NiV RNA was from a 100 µl of approximately 10^6^ cell/ml of PBMC from both, control and inoculated pigs.

### Detection of NiV genomic RNA

Total cellular RNA was isolated by TriPure isolation reagent (Roche Diagnostics) as per manufacturer's protocol. The RNA concentration and integrity was determined prior to the RT reaction. The first strand cDNA synthesis reaction was catalyzed by Superscript II reverse transcriptase (Invitrogen) with random hexamer primers. A total of 5 µl of RNA (200 ng/µl) was added to each RT reaction and incubated for 42 min at 50°C followed by 72°C for 15 min prior to amplification of target cDNA. RNase H was added to each reaction. Ten percent of cDNA synthesized in the first strand reaction was amplified on Corbett Research RotorGene RG-3000 real time system (Montreal Biotech Inc, Dorval, QC). The PCR reactions were performed with Quantitect RT-PCR kit (Qiagen), using same probe and the primers as for the NiV N-gene RT-PCR (Weingartl et al., 2005). Detection of cyclophilin mRNA was used for each sample tested as described in our protocol for mRNA cytokine expression to allow for sample comparison.

### Flow cytometry analysis of PBMC *in vitro* inoculated with NiV

#### Inoculation of PBMC with NiV

Input of PBMC per well was 10^6^ cells, and the cells were infected with 1 MOI of NiV, using protocols described for subpopulation of PBMC in previous section. The cells were harvested for analysis at 24 and 48 hrs post infection by collection from the plates and centrifugation.

#### Analysis of population frequencies

Phenotypic analysis of PBMC subsets was performed using the mAb anti-CD4 (PE conjugated, IgG2bk, BD Pharmingen), and anti-CD8 (PE conjugated IgG2ak, BD Pharmingen), and the analysis was done on separate cell preparations. The PBMC were diluted to10^6^ cells/ml, washed with MACS buffer, and pelleted by centrifugation at 300× g for 10 min. After the supernatants were discarded, the remaining cell pellets were resuspended with 1∶100 dilution of mAb against CD4+ or CD8+, or isotype controls and incubated on ice for 30 min in the dark. The cells were washed twice times with 1 ml of MACS buffer, and fixed in MACS buffer with 3.5% paraformaldehyde before removal from the BSL4, and analyzed on a two laser Flow Cytometer (Beckman Coulter, Mississauga, ON, CA). Cell populations of interest were gated based on 10,000 events with light scatter characteristics of lymphocytes. The data was analyzed using CXP software (Beckman Coulter). Infected cells incubated without any antibodies, and infected and uninfected cells incubated with isotype controls were used as negative control.

### Flow cytometry analysis of *in vivo* infected PBMC

For phenotyping lymphocytes in PBMC by flow cytometry, both single and double staining was employed to define different subpopulation. Lymphocytes were first gated from PBMC by size (FSC) and granularity (SSC). To further distinguish T lymphocytes, cells were gated for CD3+ using anti-CD3 (FITC IgG2ak, BD Pharmingen, diluted 1∶100), and the number of events collected for analysis was 10,000. The following monoclonal antibodies were used to analyse the population frequencies of T lymphocytes in PBMC: anti-CD4 (FITC conjugated, IgG2bk, BD Pharmingen), and anti-CD8 (PE conjugated IgG2ak, BD Pharmingen). Irrelevant isotype-matched antibodies were used as background controls. Unfixed lymphocytes suspensions were first filtered by pre-separation filter (Miltenyi Biotech) followed by incubation with a mixture of two antibodies directed against the surface molecule of interest. All incubations were done at 4°C for 30 min and washed twice at 600 g for 5 min. All samples were kept on ice until paraformaldhyde was added after the final wash. Analysis was on a two laser Flow Cytometer (Beckman Coulter). The data was analyzed using CXP software (Beckman Coulter).

### Statistical Analysis

Data analysis was performed using Graph Pad In Stat version 3.0 (GraphPad software, San Diego, CA). Student t-test was used for comparison between means of groups. Differences were considered as significant at P value<0.05.

## References

[1] Wang L, Harcourt BH, Yu M, Tamin A, Rota PA (2001). Molecular biology of Hendra and Nipah viruses.. Microbes Infect.

[2] Chua KB (2003). Nipah virus outbreak in Malaysia.. J Clin Virol.

[3] Hossain MJ, Gurley ES, Montgomery JM, Bell M, Carroll DS (2008). Clinical presentation of Nipah virus infection in Bangladesh.. Clin Infect Dis.

[4] Chua KB, Bellini WJ, Rota PA, Harcourt BH, Tamin A (2000). Nipah virus: a recently emergent deadly paramyxovirus.. Science.

[5] Mohd Nor MN, Gan CH, Ong BL (2000). Nipah virus infection of pigs in peninsular Malaysia.. Rev Sci Tech.

[6] Luby SP, Gurley ES, Hossain MJ (2009). Transmission of human infection with Nipah virus.. Clin Infect Dis.

[7] Middleton DJ, Westbury HA, Morrissy CJ, van der Heide BM, Russell GM (2002). Experimental Nipah virus infection in pigs and cats.. J Comp Pathol.

[8] Hooper P, Zaki S, Daniels P, Middleton D (2001). Comparative pathology of the diseases caused by Hendra and Nipah viruses.. Microbes Infect.

[9] Weingartl HM, Czub S, Copps J, Berhane Y, Middleton D (2005). Invasion of the central nervous system in a porcine host by Nipah virus.. J Virol.

[10] Berhane Y, Weingartl HM, Lopez J, Neufeld J, Czub JS (2008). Bacterial infections in pigs experimentally infected with Nipah virus.. Transbound Emerg Dis.

[11] Maisner A, Neufeld J, Weingartl H (2009). Organ- and endotheliotropism of Nipah virus infections *in vivo* and *in vitro*.. Thromb Haemost.

[12] Weingartl HM, Berhane Y, Caswell JL, Loosmore S, Audonnet JC (2006). Recombinant Nipah virus vaccines protect pigs against challenge.. J Virol.

[13] Garcia-Briones MM, Russell GC, Oliver RA, Tami C, Taboga O (2000). Association of bovine DRB3 alleles with immune response to FMDV peptides and protection against viral challenge.. Vaccine.

[14] Wong KT, Shieh WJ, Kumar S, Norain K, Abdullah W (2002). Nipah virus infection: pathology and pathogenesis of an emerging paramyxoviral zoonosis.. Am J Pathol.

[15] Lo MK, Harcourt BH, Mungall BA, Tamin A, Peeples ME (2009). Determination of the henipavirus phosphoprotein gene mRNA editing frequencies and detection of the C, V and W proteins of Nipah virus in virus-infected cells.. J Gen Virol.

[16] Mungall BA, Middleton D, Crameri G, Halpin K, Bingham J (2007). Vertical transmission and fetal replication of Nipah virus in an experimentally infected cat.. J Infect Dis.

[17] Griffin DE (1995). Immune responses during measles virus infection.. Curr Top Microbiol Immunol.

[18] Gerner W, Käser T, Saalmüller A (2009). Porcine T lymphocytes and NK cells: an update.. Dev Comp Immunol.

[19] Mathieu C, Pohl C, Szecsi J, Trajkovic-Bodennec S, Devergnas S (2011). Nipah Virus uses leukocytes for efficient dissemination within a host.. J Virol.

[20] Negrete OA, Levroney EL, Aguilar HC, Bertolotti-Ciarlet A, Nazarian R (2005). EphrinB2 is the entry receptor for Nipah virus, an emergent deadly paramyxovirus.. Nature.

[21] Negrete OA, Wolf MC, Aguilar HC, Enterlein S, Wang W (2006). Two key residues in ephrinB3 are critical for its use as an alternative receptor for Nipah virus.. PLoS Pathog.

[22] Yu G, Luo H, Wu Y, Wu J (2003). Ephrin B2 induces T cell costimulation.. J Immunol.

[23] van Reeth K, Nauwynck H (2000). Proinflammatory cytokines and viral respiratory disease in pigs.. Vet Res.

[24] Guillaume V, Contamin H, Loth P, Georges-Courbot MC, Lefeuvre A (2004). Nipah virus: vaccination and passive protection studies in a hamster model.. J Virol.

[25] Weingartl HM, Berhane Y, Hisanaga T, Neufeld J, Kehler H (2010). Genetic and pathobiologic characterization of pandemic H1N1 2009 influenza viruses from a naturally infected swine herd.. J Virol.

[26] Lo MK, Miller D, Aljofan M, Mungall BA, Rollin PE (2010). Characterization of the antiviral and inflammatory responses against Nipah virus in endothelial cells and neurons.. Virology.

[27] Virtue ER, Marsh GA, Wang LF (2011). Interferon signaling remains functional during henipavirus infection of human cell lines.. J Virol.

[28] Seo YJ, Hahm B (2010). Type I interferon modulates the battle of host immune system against viruses.. Adv Appl Microbiol.

[29] Cayrol R, Wosik K, Berard JL, Dodelet-Devillers A, Ifergan I (2008). Activated leukocyte cell adhesion molecule promotes leukocyte trafficking into the central nervous system.. Nat Immunol.

[30] Ofori-Acquah SF, King J, Voelkel N, Schaphorst KL, Stevens T (2008). Heterogeneity of barrier function in the lung reflects diversity in endothelial cell junctions.. Microvasc Res.

[31] Zachary JF, Gavin MD, Zachary JF (2007). Nervous system.. Pathologic basis of veterinary disease 4 ed.

[32] Lee BP, Imhof BA (2008). Lymphocyte transmigration in the brain: a new way of thinking.. Nat Immunol.

[33] Erbar S, Maisner A (2010). Nipah virus infection and glycoprotein targeting in endothelial cells.. Virology Journal.

[34] Weingartl HM, Sabara M, Pasick J, van Moorlehem E, Babiuk L (2002). Continuous porcine cell lines developed from alveolar macrophages: partial characterization and virus susceptibility.. J Virol Methods.

[35] Volf J, Boyen F, Faldyna M, Pavlova B, Navratilova J (2007). Cytokine response of porcine cell lines to Salmonella enterica serovar typhimurium and its hilA and ssrA mutants.. Zoonoses Public Health.

[36] Dawson HD, Beshah E, Nishi S, Solano-Aguilar G, Morimoto M (2005). Localized multigene expression patterns support an evolving Th1/Th2-like paradigm in response to infections with Toxoplasma gondii and Ascaris suum.. Infect Immun.

[37] Livak KJ, Schmittgen TD (2001). Analysis of relative gene expression data using real-time quantitative PCR and the 2(−Delta Delta C(T)) Method.. Methods.

[38] Guillaume V, Lefeuvre A, Faure C, Marianneau P, Buckland R (2004). Specific detection of Nipah virus using real-time RT-PCR (TaqMan).. J Virol Method.

